# Children^′^s physical activity and screen time: qualitative comparison of views of parents of infants and preschool children

**DOI:** 10.1186/1479-5868-9-152

**Published:** 2012-12-28

**Authors:** Kylie D Hesketh, Trina Hinkley, Karen J Campbell

**Affiliations:** 1 School of Exercise and Nutrition Sciences, Deakin University, 221 Burwood Hwy, Burwood Victoria, 3125, Australia

**Keywords:** Early childhood, Parenting, Physical activity, Screen time, Qualitative study

## Abstract

**Background:**

While parents are central to the development of behaviours in their young children, little is known about how parents view their role in shaping physical activity and screen time behaviours.

**Methods:**

Using an unstructured focus group design, parental views and practices around children^′^s physical activity and screen time (television and computer use) were explored with eight groups of new parents (n=61; child age <12 months) and eight groups of parents with preschool-aged (3–5 year old) children (n=36) in Melbourne, Australia.

**Results:**

Parents generally believed children are naturally active, which may preclude their engagement in strategies designed to increase physical activity. While parents across both age groups shared many overarching views concerning parenting for children^′^s physical activity and screen time behaviours, some strategies and barriers differed depending on the age of the child. While most new parents were optimistic about their ability to positively influence their child^′^s behaviours, many parents of preschool-aged children seemed more resigned to strategies that worked for them, even when aware such strategies may not be ideal.

**Conclusions:**

Interventions aiming to increase children^′^s physical activity and decrease screen time may need to tailor strategies to the age group of the child and address parents^′^ misconceptions and barriers to optimum parenting in these domains.

## Background

Being physically active during childhood is important for health, including the maintenance of healthy body weight, both in childhood and later life [1]. Evidence suggests physical activity and screen time behaviours are established in early childhood [2] and track over time [3,4]. Therefore, it is important that children develop healthy activity patterns early, to set them on a trajectory towards good health and healthy weight throughout life. Governments in Australia, Canada, the United Kingdom and the United States have recognised the importance of these early life behaviours by the release in recent years of national guidelines outlining optimal levels of physical activity and screen time for young children (0–5 years). Yet literature on these early life behaviours remains scarce, particularly for children prior to preschool age. For example, a 2011 review of physical activity and sedentary behaviours in children younger than three years of age identified only two studies reporting physical activity levels and only four reporting any sedentary behaviours [5]. That review called for more research to gain a comprehensive understanding of these behaviours in early life and recognised the important role played by parents.

Parents influence their children^′^s activity patterns through modelling, and provision of the social (rules and practices) and physical environments (availability of equipment for physical and sedentary pursuits) children are exposed to in the home [6]. The parental role is particularly important during a child^′^s early life, when parents are responsible for the majority of their child^′^s experiences [7]. Parental influences during this time have been shown to be associated with physical activity levels in later childhood [8]. Yet little is known about how parents view children^′^s physical activity and screen time, and their own role in shaping these behaviours during their child^′^s early life. A recent qualitative study involving parents of 5–7 year olds indicated parents believed their children to be sufficiently active and therefore did not actively promote physical activity [9].

Investigation of views and anticipated practices of parents regarding children^′^s physical activity and screen time prior to the initiation of these behaviours has not previously been undertaken. Such information would add to a meagre evidence base and provide unique insight into potential strategies to promote optimal levels of these behaviours during the important early childhood period. We hypothesise that new parents may be more receptive to strategies designed to maximise their child^′^s physical activity from its inception and limit screen time. Given this, understanding new parents^′^ views is an important first step towards designing interventions that may promote the development of active lifestyles and prevent childhood obesity. Comparing views of these new parents to those of more experienced parents may provide further insight into when and why expectations and practices may form or change, knowledge that could inform optimal times and targets for intervention. The aims of this study were: (1) to understand the views and expectations new parents have about children^′^s physical activity and screen time, prior to their infants becoming mobile, and (2) to compare these views with those of parents of preschool children, whose children have already begun establishing physical activity and screen time habits.

## Methods

Given the purpose of this research was to generate information on a previously unexplored issue, qualitative methodology was considered most appropriate. Use of this methodology allowed a truly exploratory investigation of parental views regarding children^′^s physical activity and screen time. We chose focus group methodology to enable group dynamics, inherent in the existing social groups utilised for this study, to assist in discussion initiation and development with minimal intervention by the facilitator.

### Sample

First-time parents of infants (<12 months old) and parents of preschool-aged (3–5 year old) children were recruited, September-December 2005, from one socio-economically and ethnically diverse local government area[10] in metropolitan Melbourne, Australia. First-time parents were recruited through Maternal & Child Health (MCH) centres which provide a free universal service to children aged under six years [11]. MCH nurses organise new parent groups where first-time parents can attend weekly sessions (usually for 4–6 weeks) providing information relevant to the first year of life and an opportunity to meet other new parents in their neighbourhood. All MCH centres within the local government area with new parents groups running within the time frame of the study were invited to participate (n=8) and all agreed to do so. A researcher visited each new parent group to describe the study and distribute information. Parents were invited to attend a focus group the following week at the MCH centre. Focus groups comprised 4–16 new parents with an average of eight per group.

Parents of preschool-aged children were recruited through preschools randomly selected within the local government area. To be comparable with the first-time parent groups, focus groups were sought from eight preschools. Two preschools (2/8) declined to participate and were replaced with the next preschools on the randomly ordered list. As no parents attended the focus group at one preschool, an additional preschool was recruited. Parents of children at each preschool were invited via letters distributed in their child^′^s locker/pocket/folder to attend a focus group onsite during their child^′^s preschool session. Focus groups comprised 2–9 preschool parents with an average of five per group.

All focus groups ran for 45–60 minutes, with recorded group discussions lasting for an average of 42 minutes (range 30–54 minutes). There was no correlation between group size and length of recorded discussion. This study received approval from the Deakin University Human Ethics Research Committee. All participants provided written informed consent. No incentives were offered.

### Procedure

Participants self-reported basic demographic information. All focus groups were conducted by a single facilitator, trained and experienced in qualitative research methods. As the focus groups were designed to be exploratory, no formal questions were used. Instead, participants brainstormed topics that came to mind when they thought of children^′^s physical activity and television and computer use (screen time). The facilitator recorded all topics suggested by participants and used these as prompts for discussion; she restricted further input to generic prompts and encouragement, allowing natural conversation between participants to dictate the direction of discussions. This method ensured participant- rather than facilitator-initiation of discussion content. All sessions were audio recorded and transcribed verbatim by a commercial company.

### Analyses

A grounded theory approach to analyses was used. Two researchers (KH & KC) independently reviewed the same randomly selected transcript and compared codings and themes. Each then reviewed half of the focus group transcripts, coded each quote and grouped those with similar content using the open coding method of thematic analyses [12]. A third researcher (TH) independently coded half the focus group transcripts for validation. Transcript codings were collated and themes representing the main messages conveyed by the data were extracted by two researchers (KH & TH). For validation, themes were discussed and reviewed by the third researcher (KC) and reviewed by the focus group facilitator. Sociodemographic information was summarised.

## Results

### Descriptive data

Eight focus groups were conducted with a total of 61 new parents, all mothers. The mean age of infants was 4.5 months, with most (89%) aged six months or younger. A further eight focus groups were conducted with a total of 36 parents/carers (including 1 grandparent; henceforth referred to as parents) of preschool children. The mean age of the oldest preschooler in the family (some families had multiple children in the 3–5 age range) was 4.8 years (58 months). The demographics of preschool parents were similar, but slightly more heterogeneous, to those of the new parents (Table 1). Themes from the two cohorts are discussed below. A summary of the main themes relating to physical activity and screen time, with illustrative quotes, are presented in Tables 2 and 3 respectively.

**Table 1:** Sample characteristics

	**Parents of infants**	**Parents of preschoolers**
**Characteristic**	**N (%)**	**N (%)**
**Child**		
Female gender	29 (48)	21 (58) ^††^
Age in months *[m (range)]*	5 (2–11)	58 (48–66)^††^
Only child (no siblings)	61 (100)	5 (14)
Parent in focus group*		
Female gender	61 (100)	34 (94)
Age in years *[m (range)]*	32 (21–38)	38 (28–71)
Highest level of education:		
Some high school	2 (3)	7 (19)
Completed high school	10 (16)	10 (28)
Trade certificate/apprenticeship	9 (15)	7 (19)
University degree	40 (66)	12 (33)
Employment status:		
In paid employment	10 (16)	12 (33)
On maternity leave	43 (71)	4 (11)
Home duties	8 (13)	17 (47)
Student/unemployed	0	3 (8)
Hours of paid employment per week**[m (range)]	26 (8–45)	19 (2–52)
Married/de facto relationship	60 (98)	31 (89)
English is main language spoken at home	60 (98)	30 (83)
Born in Australia	54 (89)	27 (75)
Family has a health care card^†^	5 (8)	12 (33)
Partner		
Age in years *[m (range)]*	34 (23–43)	41 (28–72)
Born in Australia	50 (83)	22 (69)
Highest level of education:		
Some high school	4 (7)	0
Completed high school	12 (20)	5 (16)
Trade certificate/apprenticeship	11 (18)	14 (44)
University degree	33 (55)	13 (41)
Partner in paid employment	55 (100)	30 (97)

^*^ Note: includes one grandparent in the preschooler group.

^**^n = 9 for parents of infants for this question; n=12 for parents of preschoolers.

^†^ Health care cards are provided by the Federal Government to low-income earners who pass a means-test. They allow access to subsidised health care, public transport, utilities and car registration.

^††^ Where participants had more than one child attending the preschool, the eldest child′s age and gender are included here.

**Table 2:** Physical activity: summary of themes from new parent and preschool parent focus groups with illustrative quotes

**Theme**	**New parent groups**		**Preschool parent groups**	
	N*	Example quotations**	N*	Example quotations**
**General impressions and expectations**				
Positive impressions and expectations regarding child becoming physically active	7	″We went to the botanical gardens on Monday. If I^′^d done that with friends we^′^d probably take a couple of bottles of wine … But when you take a child, I guess you take balls and cricket bats instead.″ [NP 8]	1	″We used to go shopping; now we go to the park.″ [PP 6]
		″It^′^s fun…they just love it, and they laugh and they enjoy that.″ [NP 3]		
		″With activity I would like her to go in as much as possible for social activities, rather than just being a loner. I think social activities provide social skills that are… a key precursor to academic success.″[NP 6]		
		″… all games in one way or another are learning; they have to learn rules, learn you can lose, you can win, you learn in a lot of respects whatever the game is.″ [NP 1]		
		″It makes me think about a bit more of a routine for us… you sit down and eat and then you go and play. So trying to introduce more structure to start with.″ [NP 2]		
		″Try and get your husband to have a bit of playtime and activity time, share it up a bit.″ [NP 2]		
		″Oh its good [physical activity]. It makes them sleep.″ [NP 4]		
		″It makes them healthy.″ [NP 2]		
Negative impressions and expectations regarding child becoming physically active	4	″It looks exhausting sometimes when you see others. Like a girlfriend^′^s got a one year old and we go out to lunch and it is exhausting because she is running after her kid and sitting down or standing up or occupied all the time. So you think it is a twenty four hour job now, but it is more when they start running around.″ [NP 2]	1	″You worry they^′^ve been at kinder all day, they^′^ve done one activity, now they^′^ve come home. Have they had enough or should they be off doing something else? Should I enrol them in so many different things? So there^′^s that dilemma.″ [PP 7]
		″Sometimes I don^′^t feel like she^′^s getting enough stimulation… she wants to run around the house and garden rather than sitting and drawing.″ [NP 7]		″You can^′^t be somewhere at four o^′^clock and somewhere at four-o-five. It^′^s just too hard. And at some point they^′^ve got to do their homework and kids need to be able to relax.″ [PP 7]
		″I don^′^t want him to be a complete sports head. I want him to have not just physical time but also times to be creative.″ [NP 5]		
		″I am thinking of my house and her just getting into all the floorboards and stuff.. I rang a friend to come over and help me spray my house because it^′^s just [I^′^m concerned about] the cleanliness issue.″ [NP 2]		
		″You just have to get used to the fact that they are going to injure themselves.″[NP 7]		
**Influences on children**^′^**s physical activity**				
Physical activity is innate in children	3	″I think it [physical activity] will come naturally for her.″ [NP 1]	2	″Some of these kids have been here for 3 hours and they^′^re running from the swing to the slide to this to that. At this age they just run naturally from one thing to another.″ [PP 7]
		″…but him being a boy and boys generally being really into sports…boys normally want to do football and basketball …″ [NP 5]		″[child 1] likes the computer and I do that with him, and he likes TV and he is much more of an inside kid. [child 2] is very different, she wants to be outside.″ [PP 8]
Family and parent activity levels influence child activity levels	4	″I just think it^′^s also influenced by the family and I know my husband grew up in a very sporty family, all boys, and it was wonderful that we were having a boy. It will just be expected of him to be an active person.″ [NP 6]	3	″Well my husband^′^s from a very sporting family so he^′^s very interested in it. He likes to watch sport on tele[vision] and we go and watch people doing sport and that sort of makes the kids interested in doing it [sport] as well.″ [PP 9]
		″Some kids are using computers and are playing Playstation all day where as some families are sport focused all the time.″ [NP 1]		″We just got a trailer bike. So now the four of us can go riding all together. For us that^′^s what we do as a family.″ [PP 8]
**Strategies for encouraging child physical activity**	5		6	
Providing a supportive environment	3	″Even having a family pet means that they have the activity; walking the dog or playing with the dog.″ [NP 3]	5	″He^′^s got the bike, he^′^s got the trampoline, he^′^s got swings, there^′^s everything there for him – scooters, rollerblades, it^′^s all there.″[PP 2]
		″We do a lot of trips away, and we tent it [camp] every year. So I^′^m really looking forward to showing [infant] the coast and taking him there and taking him to the pool, and they^′^ve got active stuff for the kids to do.″ [NP 7]		″My son loves TV and we just get to a point where we have turn it off and say, ^′^Let^′^s get dressed and go outside for a little while. You can watch it again later on.^′^ And as soon as it^′^s off they forget about it [television]″. [PP 6]
Providing supervision and companionship	2	″These playgroups are a good way to start [with active play] because we are all first mothers and there^′^s no other babies within the family, so it^′^s great for them to socialise.″ [NP 6]	6	″I find that if the neighbour^′^s kids are home, they will leave it [the television] and run next door and play over there….″ [PP 5]
				″My husband and I walk while the two kids have their bikes and they^′^ll just go off at a bit of a distance.″[PP 5]
				″If you^′^re going to do that [say they have to go outside and be active] you have to engage yourself. Sometimes they^′^re quite happy to go off but you^′^ve still got to be there.″ [PP 8]
Enrolling child in organised physical activities	3	″Getting them at an early age involved in something that^′^s once a week, you^′^d go and do this thing that^′^s physical and you keep fit.″ [NP 5]	0	
		″I^′^ve started at Gymbaroo, which is one day a week. I just see it as a bit of balance.″[NP 7]		
Parental modelling and encouragement	3	″My husband^′^s a sports nut and he can^′^t wait to get her out there. And me, I love swimming, so I can^′^t wait to get her down to the pool.″ [NP 8]	1	″Sometimes you might have to initiate things like going to the park.″ [PP 9]
		″At the moment I walk most places. That is something I would like him to be able to do as well.″ [NP 6]		
Teach fundamental movement and sports skills	0		3	″With my girls I was really keen to do things like swimming lessons from an early age… it^′^s a real advantage in school if they can do something they^′^ve sort of accomplished. Like if they get to school and they can already swim.″ [PP 9]
				″We have to go out of our way to teach them sports because we have found if you don^′^t … you get to a certain age where they just won^′^t do it… and they won^′^t play because they are the only ones who don^′^t know how to … and that kind of limits what sporting activity they do.″ [PP 5]
				″I^′^m trying to teach them activities that we used to do as children… hopscotch …and elastics.″ [PP 2]
Encouraging outdoor play/use of outdoor facilities	2	″It^′^s making sure he^′^s outside and, if you need to go to the shops, go there, do what you have to do and get home and get him outside again.″ [NP 7]	2	″We take them to the beach swimming quite regularly…if it^′^s cold we still go, they still have fun with the sand.″ [PP 1]
		″I think it^′^s pretty important that they have lots of outside time.″ [NP 8]		
**Barriers to child physical activity**	7		6	
Safety	5	″With paedophilia and people perving at your child and taking photos and abducting them in the street, or a car might hit them or anything. Like it^′^s not safe even having your child on your own front lawn unsupervised; you don^′^t know who^′^s lurking around.″ [NP 3]	2	″They [bike ride]… around the block basically. Now I live on a lane, and I am that scared of what can happen, aside from injuries. So I … watch them. But I am so scared to let them go anywhere else.″[PP 2]
		″I live across the road from the park and I think about that now. It^′^s a busy road. Would I ever let her cross?″ [NP 6]		″In daylight saving we used to ride our bike around the street at night. Our parents were just inside. See you wouldn^′^t let your children do any of these things.″ [PP 9]
		″He^′^s not allowed to play near a creek. There^′^s places he won^′^t be able to go.″ [NP 7]		
Environment	4	″I walked to school every day by myself from a very young age. And these days… I live in a street with a school in it and no one walks past my fence in the morning or the afternoon. There is not one kid who walks to school even with their parents.″ [NP 6]	1	″Now everything^′^s a lot more structured. Like you^′^ve got to enrol them in this, that and the other. Whereas we did a lot more just local…friends and out in the street, bikes and skateboards, and all that sort of stuff.″ [PP 7]
		″With the influence of today^′^s culture. Just look at all the electronic gadgets and computers and play stations and all of that. Back in our day I remember you were outside on your bike, playing hopscotch, going to the park by yourself. All of that is slowly sort of gone.″[NP 6]		
		″I know society^′^s changing. When we were young kids were out in the street all day everyday …but now you just don^′^t see that.″ [NP 3]		
		″Living arrangements [influence physical activity] as well. We don^′^t have a huge backyard and they^′^re things that I^′^m looking for now.″ [NP 5]		
Time & planning	1	″With both parents back at work and the child^′^s in childcare, there^′^s such a limited amount of time as a family. Finding regular time for any sort of team sport and playing, I think that^′^s really hard.″ [NP 8]	4	″Everything^′^s time consuming. I find myself clock watching all the time because I have a very structured routine with them.″[PP 1]
				″It^′^s scheduling it [physical activity]. It^′^s making sure that it fits in with everything else, because like I said they^′^ve got homework every night, so it^′^s all happening.″ [PP 2]
				″We^′^ll probably start that [organised sport] in the New Year. Anywhere I can take… two at the same time. It^′^s just better value for your time you know, if [child 1] and [child 2] can both do a lesson. Like we do swimming. They have back to back lessons.″ [PP 6]

^*^N refers to number of groups where theme was discussed (out of a total of 8 new parent and 8 preschool parent groups).

^**^Quotations are referred to with the notation ^′^NP^′^ for new parent groups and ^′^PP^′^ for preschool parent groups, followed by the sequential group number.

**Table 3:** Screen time: summary of themes from new parent and preschool parent focus groups with illustrative quotes

**Theme**	**New parent groups**		**Preschool parent groups**	
	N*	Example quotations**	N*	Example quotations**
**Expectations and intentions**				
Television as a babysitter	3	″I guess the only way I would see as an advantage is to stick a video on and plonk her in front of it if I had to do something for an hour. Like I think that if you^′^re at your wits end and you haven^′^t done anything all day and she^′^s been at you all day you just want peace for an hour, I^′^d put her in front of the TV.″ [NP 6]	5	″I^′^ve used the TV as a baby sitter, I^′^ll put my hand up and admit that. And I say thank god for TV…″ [PP 8]
				″That^′^s what I find with the computer games. It creates a space for you to cook dinner or to just sit.″ [PP 9]
Positive aspects of screen time	5	″… there^′^s educational [TV] programs.″ [NP 5]	5	″I like these programs because she has learnt a lot of words that I wouldn^′^t know to teach her.″ [PP 1]
		″I^′^d rather them be on a computer and know how to use a computer which is pretty much part of our society, rather than play a Playstation which is just a game … In a way they have to learn computer.″ [NP 5]		″She^′^ll say, ″Can I go and watch this on TV?″ And I^′^ll say, ″If you finish your dinner. Eat your dinner and off you go.″ Sometimes it works, sometimes it doesn^′^t.″ [PP 1]
		″Even things like Wiggles, they get up off their bum and they dance and sing. Like that^′^s better in my mind than just sitting there.″ [NP 6]		″I can get [child] to eat more easily if I stick the TV on, [and] stick the sandwiches in his hand.″ [PP 8]
		″Computers are educational aren^′^t they? It^′^s good if we start them early and it^′^s more interactive than TV.″[NP 1]		″[He] can play computer games because I feel they stimulate the imagination.″ [PP 2]
		″I think they [computers] can be educational too. The sooner they can use a computer the better for them because it is going to become their life really. Like no matter what they end up doing, they^′^re going to have to [use computers].″[NP 2]		
Negative aspects of screen time	2	″There^′^s been a lot of research on the amount of television and the amount of hours sitting in front of computer games and childhood obesity… Hopefully our generation is starting to realise the negative impact those have now.″ [NP 6]	6	″He watches TV in the morning a lot. That^′^s when all the commercials are on about McDonald^′^s and the toys. With some of the toys, he goes ″mummy quick come and have a look. I want this toy. Buy me this toy.″[PP 1]
		″They reckon the rise of ADD [attention deficit disorder] and all that, they^′^ve sort of attributed to maybe that babies growing up around TVs.″ [NP 5]		″Definitely the news, we won^′^t have the news on when the kids are there.″[PP 9]
				″I^′^ll say, ″[child] are you thirsty?″ He won^′^t even answer. He hasn^′^t even heard me ^′^cause he^′^s just mesmerized by the TV.″ [PP 6]
				″Since we^′^ve discovered DVDs, if we do that first thing in the morning then she^′^s just a different child for the rest of the day. Like if she starts off with TV, she^′^s a vegetable for the whole day.″ [PP 7]
Balance and limit setting	5	″Well you don^′^t stick your kid in front of the TV for eight hours a day sort of thing. You know maybe a little bit of TV or just balance it with more activity.″ [NP 2]	6	″I^′^ll get them out to a park for an hour or two, run them out. So when we get home they can sit down and watch a bit of TV.″[PP 1]
		″So again it just comes down to variety. You want them to be able to be a soccer player as well as manage the computer.″ [NP 6]		″I keep an eye on it (computer use). I find that I^′^ve got quite a lot of games that they do on the computer so I sort of think, she^′^s been outside, she^′^s run for two hours, some computer [is OK].″ [PP 7]
**Strategies for limiting screen time**	7		6	
Rules and limit setting	7	″I think time is one way you can possibly strike a balance. Say you can have fifteen minutes of this, or one hour of television viewing or whatever the case might be. But really it comes down to you to actually enforce that and supervise that.″ [NP 6]	6	″She knows how often she can spend on it [computer games]. It^′^s not every day anyway. She might be on the computer, maybe once a week.″ [PP 1]
		″So you^′^ve got to set rules and say, ″Okay, no television before this time in the afternoon.″ [NP 8]		″It has to be limited by time. You have to say, almost have the timer.″ [PP 9]
		″Set shows and a set amount of time, depending on their age.″ [NP 5]		
Monitoring of screen time	3	″There^′^s a place for all of those things [TV, computer games etc.], I just think it needs to be supervised more.″ [NP 7]	2	″If something like the war in Iraq comes on [the TV] we^′^ll just turn it off.″ [PP9]
Supportive environment	3	″Turn it off [TV or computer]. It gets removed.″ [NP 7]	6	″I try and get him out of the house and then when we come back we don^′^t watch TV at all.″ [PP 1]
				″My mum^′^s got a Playstation at her place so if we go there for dinner they all want to have a shot… We^′^ve literally had to unplug it some nights because they just want to sit there for hours.″[PP 6]
Encouraging alternatives to screen time	1	″You can plonk them in front of a pile of books, you can plonk them in front of blocks, you can plonk them in front of a lot of things and they^′^ll play happily.″ [NP 5]	2	″We just get to a point where we have to turn it off and say, ″Let^′^s get dressed and go outside for a little while. You can watch it again later on.″ And as soon as it^′^s off they forget about it.″ [PP 6]
				″I^′^ll just turn the TV off and I^′^ll say, ″Okay, TV^′^s off now. It^′^s play time, or let^′^s go outside, or let^′^s read a book, or let^′^s do something else.″ And I never had an issue with it.″ [PP 1]
**Barriers to limiting screen time**	4		3	
Access within the broader environment	3	″The education system is starting to enforce more and more schools to introduce computer studies and interaction with computers. So they^′^re going to have that interaction whether they have that at home [or not].″ [NP 6]	2	″They even let them take them [computers] home on weekends at primary school. My 6 year old brings it home every Friday and then they all get on it.″ [PP 2]
		″As soon as they start school, that^′^s it [they are exposed to screen time].″[NP 5]		″She has a TV in her bedroom and she sits there and watches TV and eats her breakfast in bed. So, it^′^s hard trying to get her out of the bedroom.″ [PP 3]
The influence of other carers (e.g. fathers)	2	″I cannot stand the TV being constantly on during the day and I don^′^t want the kids watching television at breakfast at all. It really makes me cross. But my husband, he^′^s like, ″But it^′^s normal for the cartoons to be on.″ There is that kind of conflict.″ [NP 8]	2	″My husband, every time he goes to the video shop, he gets them DVD^′^s and they get hooked, they want it on and on over and over again.″ [PP 1]
		″Dad plays it [Playstation] all the time. And I don^′^t want him to play it so I don^′^t know what I^′^m going to do.″[NP 5]		
		″I have enough trouble getting my husband to do that [turn the TV off], let alone my child.″ [NP 8]		
The influence of other children engaging in screen time	2	″If you^′^ve got another child down the track, if you^′^ve got older kids, and they^′^ve got shows they want to watch then you can^′^t control what the younger one are watching. Well you can but it^′^s difficult.″ [NP 5]	0	
Other commitments	1	″I think there^′^s another aspect to all of this discussion which is our lifestyle and who^′^s supervising [tv and computer time]. You know a lot of parents are working so it is going to be difficult.″ [NP 6]	2	″I think, like when you were pregnant, sometimes you have to put the video on so the others engage in that. Or if you^′^ve had a bad night. If you^′^ve been up through the night.″ [PP 9]

^*^N refers to number of groups where theme was discussed (out of a total of 8 new parent and 8 preschool parent groups).

^**^Quotations are referred to with the notation ^′^NP^′^ for new parent groups and ^′^PP^′^ for preschool parent groups, followed by the sequential group number.

### Physical activity

#### General impressions and expectations

While positive and negative impressions of children^′^s physical activity were each mentioned by one group of preschool parents, new parents devoted much discussion to these topics. All but one group discussed positive expectations they held about their child becoming physically active. These included that physical activity would assist with the promotion of learning and the development of social skills, that family activities would be shaped by child activity preferences, that physical activity conferred health benefits, it would add structure to family routines, encourage more involvement from fathers, and improve sleep. Most commonly discussed was the expectation that a child becoming physically active would increase family activity levels (discussed in four groups).

*″I think it will be a change for the better for us [child being more active]. I love being outdoors but I don*^′^*t get out as much as I should.″ [New Parent group 6].*

Four new parent groups also discussed perceived negative aspects of a physically active child. The displacement of creative and mental pursuits and the likelihood of injuries were discussed, but the predominant topic under this theme was extra parental demands. A variety of parental demands were mentioned including the pressure for parents to involve or expose their child to multiple sports and activities, the physical demands of chasing a toddler around, and the need for increased house cleaning.

″Sometimes I think there is too much pressure now on parents, but mothers in particular, to take their kids everywhere. They have to have something on every day of the week…swimming, piano, netball.″ [New Parent group 1].

One preschool group also discussed the perceived demand on parents to enrol their children in organised activities, and the dilemma of how to keep the balance between exposing them to different activities and not making them do too much.

#### Influences on children^’^s physical activity

Across both cohorts, the two influences on children^′^s physical activity primarily discussed were innate versus family influence. Three new and two preschool parent groups discussed the belief that physical activity is natural in children and will occur spontaneously, without need for direct involvement or encouragement. While not the focus of group discussions, one new parent and one preschool parent also mentioned the belief that innate activity levels and interests are different for different children, with the new parent specifically believing this is gender driven (boys are naturally ^′^sporty^′^).

″I think they have got this natural desire to run around and play, I don′t think you have to do too much to encourage them.″ [New Parent group 1].

In addition to the reflection that the drive to be physically active was innate, four and three groups of new and preschool parents, respectively, believed parent or family activity levels would influence their child^′^s activity.

″We do a bit of kayaking or cycling and sometimes they say, ′Oh we don′t want to do this…′ but as soon as we go they love it.″ [Preschool Parent group 9].

Of interest, discussions of the innate or natural nature of children^′^s physical activity and of the influence of parent or family activity levels were often intertwined within groups. These did not appear to be mutually exclusive ideas.

#### Strategies for encouraging children^’^s physical activity

Five of the eight new parent groups and six preschool parent groups discussed various strategies they intended to (new parents) or did (preschool parents) use to encourage their child to be physically active. Providing children with a supportive environment was the most commonly discussed strategy. Three new and five preschool parent groups provided various examples of supportive environments (see Table 2) including the provision of sporting equipment and pets, holidays to places that encourage active pursuits, providing rules and active alternatives to television, active transport and attending nearby schools.

″One of the priorities for me in terms of where we live will be related to school. I really will like to be close to a school so that we don′t have to rely on cars.″ [New Parent group 6].

The idea of providing companionship or playmates for their children as a strategy to encourage physical activity was also discussed in eight groups (two new parent groups and six preschool parent groups). However, unlike the similarity in discussion between the two cohorts on the provision of a supportive environment, the type of companionship discussed was different for the two cohorts. Parents of infants discussed exposing their infants to environments where they can interact with other children to foster socialisation and active play (e.g. play groups, visiting friends and cousins). Preschool parents discussed the importance of adult supervision for children^′^s physical activity and discussed companionship as often involving mothers as the ^′^playmate^′^.

″Mostly I find that I′ve got to be available. He wants to play games with me or he wants me to come out and chase him or play hide and seek. As [younger sister]′s getting older the two of them will run around and chase each other, but they usually they want me involved as well.″ [Preschool Parent group 8].

Other forms of parental facilitation were also discussed as strategies for encouraging children^′^s physical activity. These included enrolling children in organised physical activity (discussed in three new parent groups), parental modelling and encouragement of physical activity (three new and one preschool parent group), teaching their children skills (three preschool parent groups), and encouraging outdoor play (two new and two preschool parent groups). The three groups of preschool parents that discussed using physical activity to teach their children skills, talked about both fundamental movement skills and sport specific skills like swimming and batting. Discussion centred around how these skills could be drawn on as the child grew older and give them a sense of accomplishment and achievement as well as facilitating their involvement in organised sport. While two new parent groups discussed encouraging outdoor play as a strategy for encouraging physical activity, the two groups of preschool parents that discussed outdoor play focused on their use of outdoor facilities such as parks and beaches. Only one preschool parent group discussed explicitly encouraging outdoor time and this was in the context of an alternative to screen time rather than as a strategy for encouraging physical activity.

″We just get to a point where we have turn it [the television] off and say ″let′s get dressed and go outside for a little while, you can watch it again later on″, and as soon as it′s off they forget about it.″ [Preschool Parent group 6].

Other strategies for encouraging physical activity mentioned in individual new parent groups included making active play fun, getting children involved in play, exposure to different organised physical activities, encouraging self-directed play, fostering imagination, allowing freedom within a safe environment, establishing physical activity habits early, and making physical activity an important part of their life.

Within this theme, some of the individual discussion points reflected multiple strategies. For example, the following quote reflects provision of a supportive environment through active transport, as well as parental modelling and encouragement:

″We walk to school which is a 25 minute walk. The amount of complaining that we got about that initially! But now that′s just what we do. So they never even think to say can we take the car. And we always walk to the local shops and to the hairdressers and things.″ [Preschool Parent group 8].

#### Barriers to children^′^s physical activity

In addition to strategies for encouraging children^′^s physical activity, parents discussed issues they believed limited children^′^s physical activity. As observed with regard to strategies for promoting physical activity, there was variation in the consistency of views about barriers across the cohorts. Safety was the most commonly raised issue, discussed in five new and two preschool parent groups. However the focus of safety discussions differed between the cohorts. New parents referenced perceived changes since their own childhood: decreased neighbourhood safety, road safety issues such as busy roads limiting child access to parks, stranger danger and limitations they would place on their child^′^s access to neighbourhood facilities because of safety concerns. While preschool parents also referenced perceived changes since their own childhood, their discussions were focussed more generally on the need for supervision due to parents concerns for child safety in public areas.

Other issues related to structural and social environments were discussed in four new and one preschool parent group. Changes in social environments and norms since their own childhood were raised in both cohorts; new parents discussed changes in children^′^s popular culture from outdoor games to indoor electronic entertainment being the more popular past-time, and a change from walking to school to being driven as the norm. Preschool parents discussed differences over time in social norms related to independent mobility around the neighbourhood. New parents identified yard size as an issue they had not considered prior to having children, but many had small backyards that would limit physical activity options. Also noted by new parents was the difference between rural and city living, with rural environments perceived to be more conducive to physical activity, particularly from a safety aspect.

*″I grew up in a country town where you could just go off and play. There were gangs of kids running around the street playing cricket and stuff. You*^′^*re not living in the city and your kids are just free.″[New Parent group 7].*

Time and planning complexities were discussed as barriers to children^′^s physical activity in one group of new and four groups of preschool parents. Having two parents working, greater demands on family time and the planning required to engage in activities were seen as barriers to participation in structured physical activity, particularly if the family had multiple children.

*″I think it gets to a point of how much can you actually do? You know I*^′^*ve got three kids, how many places am I supposed to be after school on one night?″ [Preschool Parent group 7].*

Other barriers mentioned by both cohorts were the impact of bad weather and their limited knowledge of age appropriate activities. Additionally, new parents mentioned anticipated barriers including reduced parental influence as their child gets older, the child not enjoying physical activity, and finding activities suitable for children at varying developmental stages when there are siblings. Other barriers to engagement in physical activity mentioned by preschool parents included reduced parental energy levels, children^′^s health issues and children^′^s skill levels.

### Screen time

#### Expectations and intentions

Parents discussed both positive and negative aspects of screen time (Table 3). Positive aspects were discussed by five groups in each cohort, while negative aspects were discussed in two new and six preschool parent groups.

The most commonly discussed positive aspect of screen time was the ″babysitter″ aspect. Three groups of new parents and five groups of preschool parents discussed screen time (predominantly television time) as a means to allow parents to get things done or to have some time to themselves. Parents were quite open in discussing their practice of using screen time in this way.

″As much as I don′t like the idea of them watching a lot of television I just know I′m so exhausted at the end of the day that I′d just really like it if he would just sit and watch [television show] for half an hour while I do the dishes or whatever.″ [New Parent group 4].

″Best invention ever. I will never begrudge the television. We have a bookshelf full of videos, DVD′s you name it … Love it. It′s the best babysitter.″ [Preschool Parent group 2].

Other positive aspects of screen time discussed were: developing computer skills which will be required in later life; the promotion of physical activity through programs that encourage children to sing and dance along; the use of screen time as a ^′^reward^′^ for desired behaviour; and the educational value of television and computer use.

*″You know the other good thing about that show is they sing the same songs every day so they*^′^*re actually learning. I think that*^′^*s really good.″ [New Parent group 5].*

Negative aspects of screen time that were discussed included: detrimental health outcomes parents believed to be associated with screen time, such as obesity and attention deficit disorder; inappropriate content on television, such as violence and news reports; exposure to advertising during television viewing; and parental perception that screen time exposure causes negative emotions and temperament in their child.

″I find, with both girls, [they get] so crabby if you leave them on [computer games] for too long. I′m not sure what′s happening to their brain but it′s physical inactivity combined with mental activity. They go mad if you leave them on for too long, they get really angry.″ [Preschool Parent group 9].

The importance of balancing screen time with other activities, or setting time limits, was discussed in five new parent and six preschool parent groups.

″…hopefully we will be very, very conscious of how much time they spend with those things [electronic media], and limit it and get them outside doing things.″ [New Parent group 6].

Other topics mentioned in individual new parent groups were the difficulty of limiting exposure to electronic media, the need for parents to change their own habits to limit child exposure, diminishing parental influence on child pursuits over time, and a desire not to hinder peer relationships by restricting activities (e.g. viewing popular programs). One parent believed computer games imitating sports encouraged physical activity. Other topics mentioned by individual preschool parent groups included beliefs that: computer use was more beneficial than television; television was better for children^′^s eyes than computer exposure; morning television viewing distracted children from getting organised for school/kindergarten; television is more attractive during colder weather; and television relaxes children.

#### Strategies for limiting screen time

While many parents believed there were positive aspects to electronic media use, all but one new parent and two preschool parent groups discussed strategies for limiting their children^′^s screen time. The most commonly discussed strategy related to rules and limit setting.

″It′s all very limited. I let them watch TV when they get home for maybe half an hour until dinner′s ready. There′s no TV with dinner at all. It goes off.″ [Preschool Parent group 1].

Parental monitoring of children^′^s screen time was also seen as important by three new parents and two preschool parents groups, particularly with regard to content.

″I think the main thing is keeping an eye on that [computer use]… because there can be some negative influences on the Internet. You just want to make sure you watch what the kids are doing on there and make sure they′re not getting into anything they shouldn′t be.″ [New Parent group 3].

Further to discussions of rule setting and parental monitoring, providing a home environment that helps to limit screen time was discussed in three new parent and six preschool parent groups.

″I don′t think I′d buy my son a Playstation. I′d just avoid it.″ [New Parent group 5].

″For us it′s been not making that an option at all, we don′t have television.″ [Preschool Parent group 8].

One group of new parents and two groups of preschool parents talked about encouraging alternative activities to screen time for their children.

″You can plonk them in front of a pile of books, you can plonk them in front of blocks, you can plonk them in front of a lot of things and they′ll play happily.″ [New Parent group 5].

″We just get to a point where we have to turn it off and say, ″Let′s get dressed and go outside for a little while. You can watch it again later on.″ And as soon as it′s off they forget about it.″ [Preschool Parent group 6].

Other strategies that were mentioned but not discussed in detail were the impact of parental role modelling on children^′^s screen time (mentioned in two groups of new parents) and strategies utilised to accommodate the needs of younger children, such as not allowing older children to view programs inappropriate for younger siblings or viewing in a separate room (mentioned in one group of preschool parents).

#### Barriers to limiting screen time

In comparison to discussions relating to strategies for limiting screen time, fewer groups across the two cohorts discussed barriers. Barriers to limiting children^′^s screen time covered the broad areas of access (physical environment), the influence of other people (social environment), and other demands on parents.

Three new and two preschool parent groups discussed access to electronic media within the broader environment as a potential barrier to limiting children^′^s screen time. Two of these new parent groups and one preschool group talked about the school environment and the prevalence and importance of computers as a barrier to limiting children^′^s computer use at home. These groups, and one group of preschool parents, also discussed the ready access to computers and ″electronic gadget culture″, as a further barrier. The other new parent group discussed barriers within the family environment such as readily accessible electronic games.

″From when I was a child, the level of exposure to that type of [electronic] media has just skyrocketed. Like kids are so computer literate and exposed to it at such an early age. And so much of it!″ [New Parent group 5].

Barriers related to the social environment and the influence of other people also received considerable discussion. The influence of other carers, particularly fathers, who like television or electronic games themselves was discussed in two new parent and two preschool parent groups. Similarly the difficulty of restricting screen time when other children, such as peers, siblings or cousins, are engaging in these activities was discussed in two new parent groups.

″I have enough trouble getting my husband to do that [turn the TV off], let alone my child.″ [New Parent group 8].

″Like our nephew who is ten … plays … computer games. So we′ve also got that element in the family. With grandma [saying], ″Oh, but [cousin] always did that. Why can′t your [child play computer games]?″ [New Parent group 8].

Parental factors were discussed by two groups of preschool parents and one group of new parents as further potential barriers to limiting screen time. These parental factors included other commitments, such as work and caring for other children, lack of parental energy, and lack of parental time. Within these discussions the use of television as a babysitter (also discussed above under the theme of positive aspects of screen time) was also infused. Parents discussed the use of television to allow them time to get other things done.

″I′m not a big television fan at all. But I have used it when we had our restaurant and I′d be working twelve hours. I would put him there [in front of the TV], with his breakfast and I′d get another hour [sleep]. I hated myself for doing it but I needed it in order to function for the rest of the day, and then go to work.″ [Preschool Parent group 8].

## Discussion

This study sought to describe the views and expectations of new parents of infants regarding their child^′^s future physical activity and screen time, and to compare these to parents of preschool children already engaging in such activities. Increased knowledge of parenting in regard to these behaviours during early childhood is important to understand how best to support parents to promote increased physical activity and reduced screen time in children, with the ultimate aim of preventing childhood obesity and other adverse outcomes.

Overall, regardless of their child^′^s age, parents believed children are naturally physically active and thus there is little need for parent engagement. For example, few parents considered the need to promote outdoor play, despite this being one of the most consistent correlates of physical activity in young children [13]. It is apparent that such ideas do not change as children become older and parents have more first-hand experience of children^′^s physical activity. This finding corresponds with results of a previous qualitative study where parents reported not actively promoting their 5–7 year olds to be physically active due to the belief they would naturally achieve sufficient levels of physical activity [9]. The belief that children are innately active is at odds with evidence showing even young children spend a very small proportion of their time being physically active [14-16]. The lack of physical activity observed in young children, despite the commonly held belief that children are naturally active, may stem from another issue raised in this study, the fact that constraints are placed on children^′^s physical activity both by parents (e.g. due to safety concerns) and the environment (e.g. smaller backyards). The social changes parents in this study noted since their own childhood are likely to have increased the level of constraint placed on contemporary children^′^s physical activity and may contribute to the low levels of physical activity observed. The misconception about children being naturally physically active has important implications for the development of strategies addressing children^′^s physical activity. If parents do not believe this is an area they need to actively encourage, it seems unlikely that they will engage in strategies to promote physical activity. Thus interventions aiming to increase the activity levels of children may need to first convince parents, not only of the importance of physical activity, but of the fact that children do not naturally achieve enough physical activity for optimal health. Only then is it likely that parents will see this as an important part of their role.

While parents shared their overarching views about child physical activity screen time, the consideration of strategies to promote or limit (respectively) these behaviours differed by cohort, suggesting a change in parental expectations as children age. This may be due to the different experiences and current circumstances of parents across the cohorts. Issues discussed by parents of preschool children may not yet have been encountered by parents of infants, suggesting the importance of understanding later life stages and considering anticipatory guidance approaches in interventions. While most new parents were optimistic regarding their ability to positively influence their child^′^s physical activity and screen time, such optimism was not apparent amongst parents of preschool children. Parents of preschool children generally spoke with some resignation, noting that they used strategies they knew were not ideal (e.g. television as a babysitter) because it was effective for them. Similar findings have been reported in quantitative research on parental self-efficacy to promote obesity protective behaviours amongst 18 month and 5 year olds, published since this study was conducted [17]. In that quantitative study, self-efficacy was higher amongst parents of younger children and was directly associated with child behaviours. For example, parents demonstrating greater self-efficacy for limiting television viewing had children who watched less television [17]. This highlights the importance of parental confidence for shaping desired behaviours and of providing parents with strategies early on, to manage the increasing demands of parenting as children get older and family circumstances change.

Further differences between the cohorts were seen in the consideration of barriers to facilitating physical activity and limiting screen time. Parents of preschool children more frequently identified a range of issues, such as restricted time and the impact of external influences. It is likely that parents of preschool children may be more time poor that those of younger children, being more likely to have returned to work (either at home or outside the home) and more likely to have additional children to care for. However some identified barriers could be overcome by providing parents with strategies and support. For example, inclement weather was identified as a barrier to promoting physical activity and limiting screen time. Providing parents with ideas for active indoor pursuits (e.g. dancing), and access to low-cost local community resources such as playgroups and indoor play centres may be useful strategies to overcome this barrier. Further, activities requiring minimal supervision such as dancing could act as an alternative to the use of television as a ^′^babysitter^′^ for time poor parents.

Interestingly, parents of infants identified more positive than negative aspects of screen time, with television viewing in particular seen as a pastime with many benefits. This is despite emerging evidence that television may have harmful consequences for children in the short and longer term. Exposure to television has been associated with obesity [18], lipid abnormalities [19], sleep problems [20], attention problems [20], aggressive behaviour [20], externalising behaviours [20], emotional reactivity [20], and poorer reading ability [21], in children, and childhood viewing has being associated with lower educational attainment in adulthood [22]. A longitudinal study spanning 5 to 26 years of age found excessive television viewing during childhood was associated with increased risk of overweight, raised serum cholesterol, poorer cardiorespiratory fitness, and increased risk of smoking [23]. Further, level of television viewing has been shown to track, even from as young as two years of age [24], suggesting a need to limit television from early in life. In addition to limiting television viewing, parents could be supported to understand the importance of circumscribing the content and mode of viewing. Co-viewing (parents watching with the child and discussing content) may confer social benefits and has been shown to improve any positive effect from beneficial content [25].

While this study sought to investigate the views of parents, those who attended the focus groups were predominantly mothers. Only two fathers were included, both in preschool groups. This reflects a limitation of the sampling technique and likely reflects the fact that mothers continue to be the primary caregivers, particularly for infants and young children. Nonetheless the views of fathers with regard to children^′^s physical activity and screen time may provide alternative insights into these behaviours and warrant further investigation.

Other aspects of the sample also reflect limitations of the study. As the focus groups were conducted during the day, working parents were underrepresented; the sample was also predominantly dual-parent, Australian-born and English-speaking. Despite sampling from a socioeconomically diverse local government area, there was greater heterogeneity in socioeconomic characteristics, such as level of education and low income health care card, in the preschool parent groups than in the infant parent groups. Such sample characteristics may limit the generalisability of the findings but do not impact internal validity.

In considering these findings it should be noted that as discussions were unstructured and topics were initiated by the group, not all topics were covered in all groups. Further, the length and depth of discussion relating to individual topics varied substantially between groups and between topics, there were a large total number of themes discussed, and some topics were touched on only briefly in some groups. As new parents^′^ beliefs about physical activity and screen time is a previously unexplored area, there was no evidence with which to inform structured questions for qualitative investigation. Thus an unstructured format was the most appropriate methodology for exploring the views and expectations of new parents. The unstructured approach was a strength of this study, given discussion was parent initiated rather than directed by preconceived notions of the researchers. Further, as physical activity and screen time beliefs of new parents have not previously been explored, they provide unique data with which to inform future research in this area. A further strength of this study was the novelty of comparing views of parents temporally, providing unique insights into ways parents^′^ thinking about these behaviours evolves, and when it may be most effective to intervene to support parents to promote physical activity and limit screen time in their children. The information gleaned from this exploratory study raises additional questions that warrant further research, for example, how the attitudes and intentions of parents of preschool aged children have changed and evolved since their children were infants, and how they have changed with the addition of siblings and with changed parental circumstances (e.g. returning to work).

## Conclusions

In conclusion, this study provided unique insight into the views of parents of young children on their child^′^s physical activity and screen time and their own involvement in influencing these behaviours. While many views were shared by new parents of infants and parents of preschool-aged children, new parents appeared more excited and optimistic about their role in shaping these behaviours. Interventions to increase young children^′^s physical activity and reduce their screen time may benefit from addressing the erroneous parental perception that children innately engage in sufficient physical activity for health. Further, such interventions could be enriched by understanding barriers to engaging in physical activity and limiting electronic media use and aiming to provide practical strategies and skills to allow parents them to overcome these barriers. Such skills and strategies may need to be tailored to the stage of parenting. In comparison to new parents of infants, who are optimistic about their abilities to influence their child^′^s behaviours and have not yet encountered the barriers to promoting physical activity and limiting screen time, parents of older children may require additional strategies and motivation to overcome the barriers they have already experienced.

## Competing interests

The authors declare that they have no competing interests.

## Authors’ contributions

KH and KC conceived the study and oversaw its conduct. KH and KC reviewed the focus group transcripts and applied thematic codings. TH coded half of the focus group transcripts for validation. KH and TH extracted themes from the data codings and drafted the results section of the manuscript. KH drafted the manuscript. KC and TH reviewed the manuscript. All authors read and approved the final manuscript.
